# Beneficial Perioperative Aspects Favor the Use of Percutaneous Crossed Pinning over Antegrade Nailing in Pediatric Supracondylar Fractures—A Retrospective Comparative Study

**DOI:** 10.3390/children10050830

**Published:** 2023-05-02

**Authors:** Frederik Greve, Peter Biberthaler, Christoph Castellani, Georg Singer, Holger Till, Helmut Wegmann

**Affiliations:** 1 Department of Trauma Surgery, Klinikum Rechts der Isar, Technical University of Munich, 81675 Munich, Germany; 2 Department of Pediatric and Adolescent Surgery, Medical University of Graz, 8063 Graz, Austria; 3 Department of Trauma Surgery, RoMed Hospital Wasserburg am Inn, Gabersee 1, 83512 Wasserburg, Germany

**Keywords:** pediatric supracondylar humeral fracture, percutaneous crossed pinning, antegrade nailing, pediatric fractures

## Abstract

(1) Background: Displaced supracondylar humeral fractures in pediatric patients can be treated by either antegrade nailing (AN) or percutaneous crossed pinning (PCP). The aim of this study was to compare the intra- and perioperative management, complications and outcome of AN and PCP. (2) Methods: This retrospective study enrolled 271 individuals (median age 5 years, IQR 4–7 years) who underwent AN (*n* = 173) or PCP (*n* = 98). Patient history was analyzed for incidence of nerve injuries, postoperative treatment, postoperative malrotation, time of hospital stay, time to implant removal and revision rate. Operative procedures were investigated for duration and radiation exposure. (3) Results: PCP was associated with a significantly lower radiation exposure (dose area product: PCP mean 20.1 cGycm^2^ vs. AN mean 34.7 cGycm^2^, *p* < 0.001; fluoroscopy time: PCP mean 1.1 min, range 0.1–8.1 min, vs. AN mean 1.5 min, range 0.1–7.1 min, *p* < 0.001), duration of surgery (PCP mean 32.2 min vs. AN mean 48.3 min, *p* < 0.001) and time to implant removal (PCP mean 37 days vs. AN mean 113 days, *p* < 0.001). Cast removal was performed earlier in the AN group (PCP mean 30.2 days vs. AN mean 20.4 days, *p* < 0.001) and there were fewer iatrogenic nerve lesions (PCP: 24% vs. AN: 8%, *p* < 0.001). (4) Conclusions: In the investigated study population, the analyzed parameters seem to favor the use of PCP. The advantages of AN should be weighed against its drawbacks. For special indications, AN remains a relevant technique in supracondylar fracture treatment, and surgeons should be familiar with this procedure.

## 1. Introduction

With an incidence of 177.3 per 100.000, supracondylar humeral fractures are commonly encountered in pediatric patients [1] and account for 55% to 80% of all elbow fractures [2]. These non-articular distal metaphyseal fractures usually occur within the first decade of life with a peak between 6 to 10 years of age. The fracture rate for boys is up to 1.6 times higher than that for girls [3]. The internationally used Gartland classification was introduced in 1959 and distinguishes three fracture entities based on the degree of displacement: type 1, non-displaced; type 2, moderately displaced; and type 3, severely displaced. In 2006 a Gartland type 4 was added which describes an unstable fracture in flexion and extension due to anterior and posterior periosteal disruption [4,5]. 

Non-displaced or minor displaced Gartland type 1 and 2 fractures without rotational malalignment can be treated conservatively by the cuff-and-collar method [6]. 

**Figure 1:**
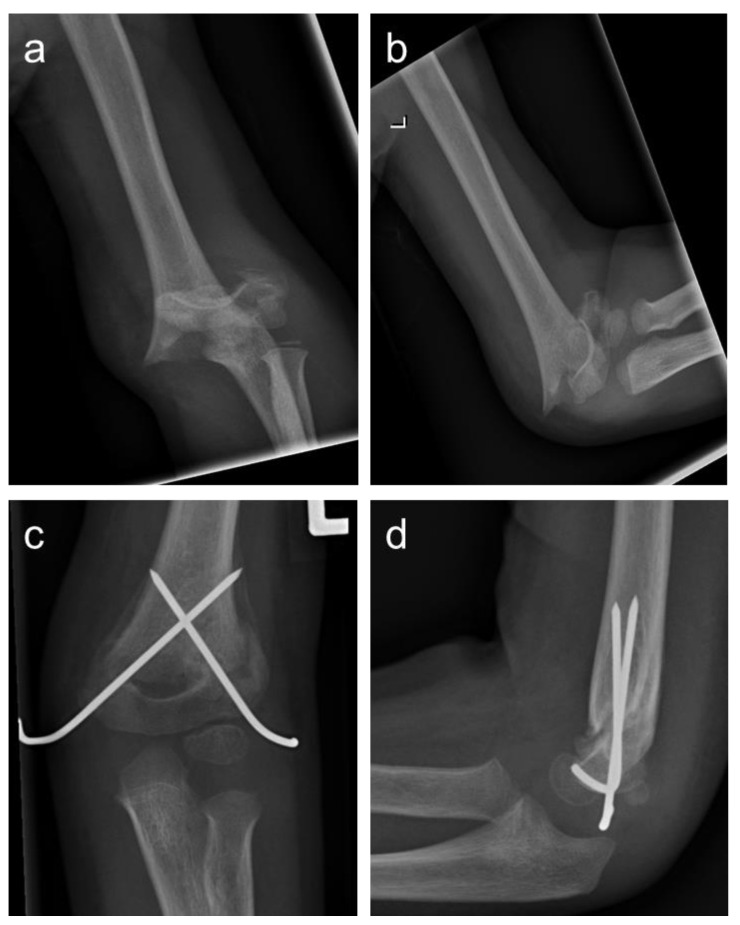
Anterior–posterior and lateral radiographs of a Gartland type 3 supracondylar humeral fracture (**a**,**b**); the patient was treated with PCP using 2 Kirschner wires (**c**,**d**).

For displaced supracondylar humeral fractures, however, the preferred treatment method is closed reduction and percutaneous crossed pinning (PCP) (Figure 1) [7,8]. Commonly described complications of this procedure are iatrogenic nerve lesions in up to 20% [9], infections and joint stiffness [10,11]. As an additional disadvantage, the postoperative protocol consists of cast immobilization for a minimum of 3 to 4 weeks depending on radiological signs of consolidation. 

**Figure 2:**
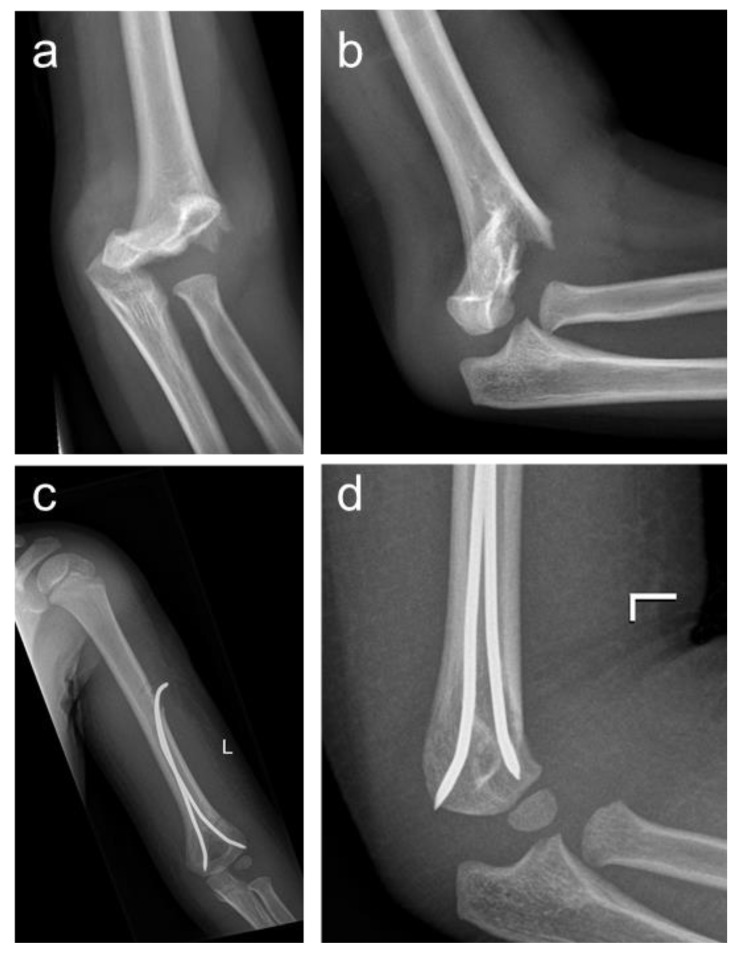
Anterior–posterior and lateral radiographs of a Gartland type 2 fracture (**a**,**b**); antegrade nailing was performed using 2 elastic nails (**c**,**d**).

To challenge these drawbacks, Prévot and coworkers introduced the antegrade nailing (AN) technique (Figure 2) in 1990 [12]. This minimally invasive method allows early postoperative elbow motion because the postoperative protocol does not require mandatory cast immobilization. Regarding the outcome, AN is associated with favorable morphological and functional results [13,14]. Furthermore, it was found to be superior to PCP in terms of iatrogenic nerve lesions, and it has been shown to prevent postoperative cubitus valgus and varus deformity in the case of impacted column fractures [8,11,13,15]. Despite these advantages, AN has not yet been established as a routine procedure in the treatment of displaced supracondylar fractures, and the literature regarding this alternative surgical procedure is still sparse. As far as we know, apart from a biomechanical investigation [16] and one clinical investigation with a focus on postoperative outcome [13], there is no clinical comparative study of PCP and AN.

To elaborate so far not investigated advantages and disadvantages of PCP and AN, the aim of this retrospective study was to compare both techniques in terms of intraoperative management (time of surgery and radiation exposure), complications and postoperative management in a consecutive series of pediatric and adolescent patients with displaced supracondylar humeral fractures. The results should facilitate the selection of the available surgical techniques.

## 2. Materials and Methods

In this retrospective single-center study, all pediatric and adolescent patients operatively treated with either AN or PCP for displaced supracondylar humeral fractures during a nine-year period were identified from the patient data management system. The selection of the respective surgical procedure was performed according to the surgeons’ preference. Exclusion criteria were open fractures and combined humeral and forearm fractures. The fractures were classified according to the Gartland classification [4]. Only Gartland type 2 and Gartland type 3 fractures were analyzed in this study.

**Figure 3:**
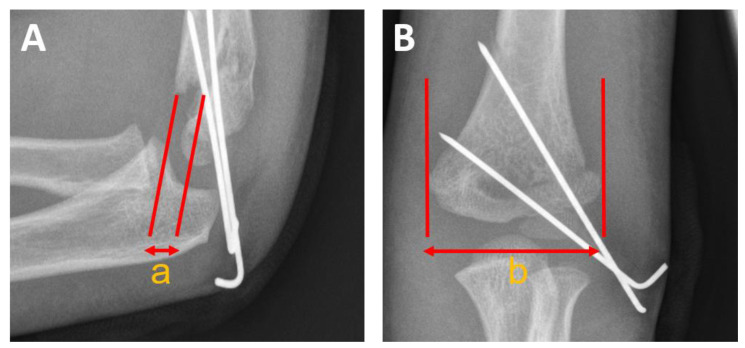
Calculation of the von Laer malrotation quotient (rfq). (**A**) Dimension of the rotational spur in millimeters ‘a’. (**B**) Dimension of the distal fragment ‘b’ in the anterior–posterior plane. For calculating the von Laer malrotation quotient (rfq), ‘a’ is divided by ‘b’ (a/b) [18].

Information concerning general information (age, gender, pre- and postoperative nerve injuries and rehabilitation protocols) was extracted from the patients’ medical histories and documentation of the routine follow-up examinations. Furthermore, the need for revision surgery, time of hospital stay, time of postoperative splint immobilization and time to implant removal were assessed. The surgical reports were analyzed for duration of the surgical procedure, fluoroscopy time and dose area product (DAP) exposure in cGycm^2^. Postoperative radiographs were analyzed for malrotation. The von Laer malrotation quotient (rfq) was used to quantify the dimension of the rotational spur as an indicator for malrotation (Figure 3) [17]. The study was approved by the institutional ethics committee (29-094 ex 16/17, date of approval: 12 December 2016).

### 2.1. Statistical Analysis

All data were managed with Microsoft Excel 2018 (Microsoft Corporation, Redmond, WA, USA). For statistical analysis, data were transferred to GraphPad Prism Version 9 (Graphpad Software, Boston, MA, USA). Nominal and ordinal data are presented as numbers and percentages; metric data are presented as means, standard deviation (SD) and range. In the case of not normally distributed data, median and interquartile range were used for data description. Normal distribution was tested by use of the D’Agostino and Pearson test.

The chi-squared test was used to compare categorical variables such as presence, location and quality (sensory, motoric or sensomotoric) of postoperative nerve lesion; need for postoperative cast immobilization; and revision surgery. The Mann–Whitney U-test was chosen for the comparison of metric data (follow-up, duration of the surgical procedure, fluoroscopy time and dose area product (DAP) exposure, von Laer malrotation quotient, duration of hospital stay, and postoperative immobilization). *p*-values of <0.05 were considered statistically significant. Correction for multiple testing was performed by the Holm-Šídák method.

### 2.2. Surgical Technique

#### 2.2.1. Closed Reduction

Patients underwent general anesthesia and were positioned in a supine position. The injured arm was placed perpendicular to the operating table on a radiolucent arm table. The image intensifier was positioned below the table.

In the vast majority of cases, closed reduction was performed by a combination of gentle longitudinal traction with varus or valgus stress for reduction in the frontal plane and flexion and pronation for correction in the sagittal plane. More complicated fractures needed additional repositioning maneuvers. The reduction was then visually verified on an anterior–posterior and lateral image under persisting flexion. Criteria for an acceptable reduction were physiologic Baumann angle, humeral–ulnar angle, intact medial and lateral column and the anterior humeral line passing the middle third of the capitellum.

#### 2.2.2. Retention by Antegrade Nailing (AN)

The proximal entry point is located at the tuberosity of the deltoid muscle at the lateral humerus shaft. Following a small incision and preparation to the bone, the near cortex was drilled or opened with an awl for perforation to access the medullary canal. Two pre-bent elastic nails of 1.6 mm or 2.0 mm diameter (depending on age, weight and diameter of the of the humeral marrow cavity) were inserted to the fracture zone and gently advanced into the distal fragment while fracture reposition was secured. The distal nail was rotated 180° toward the medial column for divergence for maximum biomechanical stability. The nail tips were finally impacted into the radial and ulnar column of the supracondylar area. After radiological verification of the osteosynthesis, the proximal nail tips were cut near the bone to avoid irritation of the deltoid muscle. Cast immobilization is not necessary after AN. Patients were allowed to move without restrictions. Nail removal was performed under general anesthesia after consolidation.

#### 2.2.3. Retention by Percutaneous crossed Pinning (PCP)

Following repositioning, two Kirschner wires (K-wires) of 1.6–2 mm diameter were inserted. The first K-wire was inserted from the lateral radial side and was placed in a ventral/dorsal trajectory. The second K-wire was inserted slightly ventral of the ulnar epicondyle for prevention of iatrogenic ulnar nerve injury in a dorsal/ventral trajectory. The K-wires were advanced across the fracture zone and crossed in the proximal fragment for obtaining maximum stability. After impaction in the far cortex, proximal ends were cut. The ends protrude from the skin for easy removal after consolidation. Only lateral pin insertion and mini-open surgery as an adaption of the described technique were not performed in the investigated study population.

Patients treated with PCP needed postoperative immobilization in 60° flexion, and weight bearing was not allowed. With very few exceptions, K-wire removal was performed in sedoanalgesia without the need for general anesthesia.

## 3. Results

### 3.1. Study Population

During a nine-year period, 304 pediatric and adolescent patients were surgically treated for displaced supracondylar humeral fractures. The exclusion criteria were met by 33 patients which were not considered in the further analysis. Therefore, 271 patients with a median age of 5 years (IQR 4–7 years, range 1–16 years) were finally enrolled. Of these, 173 patients (63.8%) were treated with AN and 98 patients (36.2%) were treated with PCP. A preoperative X-ray was retrospectively available in 192 cases (70.8%; 192/271). Patients of the PCP group presented for routine follow-up examination after a median of 2 months (IQR 1–4 months), and patients of the AN group presented for routine follow-up examination after a median of 3.5 months (IQR 2–5 months). Details of the study population are depicted in Table 1.

### 3.2. Preoperative Nerve Lesions

Preoperative nerve pathologies were diagnosed in 24 (8.9%; 24/271) of all cases. However, lesions were only detected in patients with Gartland type 3 fractures (*p* = 0.038). In more than half of the cases, the median nerve (62.5%; 15/24) was affected, followed by the ulnar nerve (20.8%, 5/24). A lesion of the radial nerve was detected in only one patient (4.2%; 1/24). A combined lesion of median nerve/radial nerve, median nerve/ulnar nerve, and a combination of all three nerves was detected in one case each. While a sensomotoric lesion (62.5%; 15/24) was diagnosed in 15 cases, a pure sensory impairment (37.5%; 9/24) was detected in 9 cases. Ten (41.7%; 10/24) patients with a preoperative nerve lesion were treated with PCP, and 14 (58.3%; 14/24) received AN. 

### 3.3. Surgery Duration and Radiation Exposure

PCP was associated with a significantly shorter duration of surgery, fluoroscopy time and DAP compared to AN (Table 2). Hospital stay was significantly shorter in the PCP group (2.4 days; SD 0.98, range 1–6) compared to the AN group (2.7 days; SD 1.2, range 1–9) (*p* < 0.05).

### 3.4. Postoperative Nerve Lesions

Iatrogenic postoperative nerve pathologies were seen in 38 patients (14%; 38/271), with significantly (*p* < 0.01) more lesions in the PCP group (24.5%, 24/98) compared to the AN group (8.1%; 14/173). The distribution of iatrogenic postoperative nerve lesions is summarized in Table 3. Motoric impairment was shown by 65.8% (25/38) of the iatrogenic nerve lesions, while 34.2% (13/38) were only sensory. All lesions were diagnosed postoperatively, and the patients did not present these lesions preoperatively. All cases of iatrogenic nerve injury resolved without surgery within 6 months. 

### 3.5. Postoperative Malrotation

Signs of postoperative malrotation were observed in 21 patients (21.4%, 21/98) of the PCP group and in 45 patients (26%) of the AN group. There was no statistical difference regarding the extent of postoperative malrotation according to the von Laer malrotation quotient (rfq PCP: median 0.12, IQR 0.1–0.15; rfq AN: median 0.13, IQR 0.08–0.16; *p* = 0.787). 

### 3.6. Rehabilitation Protocol and Cast Removal

Postoperative immobilization was carried out in all patients treated with PCP (98/98; 100%) and in 48 (27.7%; 48/173) of the patients treated with AN (*p* < 0.01). In the case of postoperative immobilization, the cast was removed after a mean of 30.2 days (SD 14, range 10–114) in the PCP group and after a mean of 20.4 days (SD 7.8, range 6–40) in the AN group (*p* < 0.01).

### 3.7. Revision Surgery

Revision surgery due to secondary displacement or implant malposition was necessary in 12 out of 271 patients (4.4%). Two of these complications occurred in children treated with PCP (2%; 2/98), and 10 occurred in children treated with AN (5.7%; 10/173,). This distribution was not significantly different (*p* = 0.392).

### 3.8. Implant Removal

The time to implant removal was significantly shorter in the PCP group (median 31 days; IQR 26–39 days) compared to the AN group (median 96 days; IQR 69–137 days, *p* < 0.01). In the PCP group, almost all implant removals (94.9%; 93/98) could be performed in an outpatient setting under sedoanalgesia. In five patients, the K-wires had to be removed under general anesthesia. All implants in the AN group were removed under general anesthesia, and 24 (13.9%; 24/173) patients were hospitalized for more than one day. There were no complications associated with implant removal.

## 4. Discussion

In this study, we have compared AN with PCP for the treatment of supracondylar humeral Gartland type 2 and 3 fractures in pediatric patients. The findings of this clinical comparison seem to relativize the proclaimed benefits of the AN procedure. PCP was found to require less radiation exposure and less operation time with a shorter hospital stay and no long-term complications but showed a higher prevalence of clinically transient postoperative ulnar nerve pathology. 

Despite the potential benefits of AN as an alternative technique in the treatment of pediatric supracondylar fractures, PCP is still the gold standard [1,13,19,20,21]. For the elaboration of potential reasons, this investigation serves to identify the advantages and disadvantages of the respective surgical techniques. 

The average age of around 5 years of the investigated patient collective is slightly younger than the reported epidemiology which describes a peak around 6 to 10 years [1,22,23]. Preoperatively, almost 9% of the patients presented with posttraumatic nerve lesions, of which all were seen in Gartland type 3 fractures. This is slightly below the previously reported prevalence (12–20%) of immediate posttraumatic nerve injuries [24]. Confirming the results of previous reports, all diagnosed posttraumatic nerve injuries of our study population recovered to full function after the application of the wait-and-see strategy [25,26]. 

One of the most important proclaimed advantages of AN is a minimized risk for postinterventional nerve injury. In our collective, we were able to confirm a higher likelihood of iatrogenic nerve injury in the PCP group. The prevalence of almost one-quarter was even slightly higher than the reported 20% in the literature [9]. Fortunately, none of the patients suffered from persisting nerve impairment during the routine follow-up examinations. Nevertheless, ulnar nerve lesions remain a common complication during PCP [13]. To minimize the risk, a mini-open incision over the ulnar epicondyle to palpate the ulnar groove might facilitate correct pin placement [27,28]. In our collective, the pins were drilled percutaneously without a mini-open incision, which might explain the relatively high incidence of transient ulnar nerve lesions. An additional mini-open incision could potentially decrease the rate of iatrogenic nerve lesions. As a further alternative, pure lateral pin fixation could reduce the risk of nerve injury [29,30]. Recent investigations describe similar functional and radiological outcomes of PCP and lateral fixation [31,32], while loss of torsional stability seems to be a problem after lateral fixation [21,33]. 

Regarding iatrogenic nerve impairment, we were able to confirm a lower risk for nerve lesions after AN, but the advantage could lose its impact if described adjustments of the PCP technique are applied.

As a potential benefit in contrast to PCP, cast immobilization is usually not necessary after AN, with consecutive shorter immobilization time in our collective. Surprisingly, about one-quarter of our patients received postoperative cast treatment. Indications were pain, swelling and additional stability in case of more complex fractures. All PCP patients underwent postoperative immobilization, and none of these developed any long-term complications despite this additional restriction. Hence, we advocate that the proclaimed advantage of no cast immobilization after AN is not striking according to our data.

Furthermore, PCP and AN seem to be equal in need for revision surgery and prevalence of postoperative malrotation. 

A common drawback of AN is reported by Weinberg et al. who describe the method as technically demanding [11]. Despite the experience with this technique in our center, the mean duration of surgery was significantly longer in the AN patients. This is reflected by a significantly longer fluoroscopy time and higher DAP. This extensive radiation exposure might derive from the longer surgery time and the increased need for intraoperative radiographs during the procedure. However, there are also technical aspects that lead to higher radiation exposure. To obtain an appropriate radiological overview, the aperture of the image intensifier must be opened to a wider cross-section. Furthermore, in the presence of multiple implants—such as long elastic nails as used during AN—the radiation intensity automatically adjusts to a higher level. This is also a long-term threat for the surgery team being frequently exposed to higher radiation intensity. Despite statistically significant results, one has to question critically if a mean difference of 0.4 min longer fluoroscopy time is of clinical importance. Nevertheless, we focus on a pediatric study population, and we believe that every potential aspect of radiation protection is worth mentioning.

Another major drawback of AN is the need for general anesthesia during obligatory implant removal. In the majority of PCP patients, K-wire removal was possible under sedoanalgesia in an ambulant setting. In the AN group, 14% of the patients were hospitalized for more than one day. Besides inconvenience for the patients, the economic impact has to be considered. 

The shorter time to implant removal in the PCP group appears to be of minor relevance as the descending nails in the AN technique are positioned subcutaneously in the region of the deltoid muscle. Thus, the implants do not influence patient comfort. 

It is important to note that this study is not without limitations. The main limitation is the retrospective character of the study. A randomized allocation of the performed surgical procedure was not possible. AN or PCP was chosen according to the surgeon’s preference, which might bias our results. On the other hand, this enables high performance quality and standardization as the surgeons are experienced with the selected technique. Data were allocated with the best possible care, and the high number in both groups and the well-documented and standardized postoperative follow-up led to a high data quality despite the retrospective study design.

Another limitation is the enrollment of very young patients under 3 years of age which limits the quality of the neurological examination. Data assessment relied on the combination of a clinical neurological examination and reports from the parents. While motoric lesions could be assessed reliably in all patients, sensory impairment might have been overlooked in very young patients.

As already reported by previous studies [8,11,13,14,15,18,30,32,34], the assessment of clinical and functional outcome was not the aim of this study. Therefore, no study-specific clinical follow-up examination including assessment of outcome scores, apart from routine postoperative follow-up, was performed.

## 5. Conclusions

Compared to PCP, AN proved to be safer in terms of iatrogenic nerve injury, allowed earlier postoperative mobilization and was equal with respect to the need for revision and postoperative malrotation. However, the duration of surgery, fluoroscopy time, DAP and postoperative hospital stay were significantly increased in patients treated with AN. The main drawback of AN is the need for a second general anesthesia for implant removal. As none of the iatrogenic nerve injuries in both groups required revision surgery, and postoperative immobilization in young patients does not lead to long-term sequelae, the advantages of AN must be weighed against the drawbacks. Taking into account the lower level of evidence provided by the retrospective study design, our data seem to indicate that PCP might be the preferred technique in terms of shorter surgery time, shorter hospital stay and less radiation exposure at the cost of transient ulnar nerve irritations which might be prevented by a mini-open approach. However, the data are not sufficient to definitely prefer one procedure over the other. For a comprehensive coverage of treatment options, surgeons should be familiar with AN as an alternative technique.

## Figures and Tables

**Table 1:** Characteristics of the study population regarding age, gender, Gartland classification and follow-up.

	Antegrade Nailing (AN)	Percutaneous Crossed Pinning (PCP)
**Patients**	*n* = 173, 63.8%	*n* = 98, 36.2%
**Age (years)**	5 (IQR 4–6)	6 (IQR 4–7)
**Gender (m:f)**	*n* = 92, 53.2% *n* = 81, 46.8%	*n* = 55, 56.1% *n* = 43, 43.9%
**Gartland (2/3)**	2:*n* = 27, 22.9% 3:*n* = 91, 77.1%	2:*n* = 13, 17.6% 3:*n* = 61, 82.4%
**Follow-up (months)**	2 (IQR 1–4)	3.5 (IQR 2–5)

Data are displayed as counts and percentages or mean ± standard deviation and range or median and interquartile range.

**Table 2:** Comparison of surgery duration and radiation exposure.

	AN	PCP	*p*-Value
**Duration of surgery (min)**	48.3 ± 25.2 (19–150)	32.2 ± 22.6 (5–125)	<0.01
**Fluoroscopy time (min), available in *n* = 247**	1.5 ± 1.3 (0.1–7.1)	1.1 ± 1.3 (0.1–8.1)	<0.01
**DAP (cGycm^2^), available in *n* = 169**	34.7 ± 39.2 (2.4–223)	20.1 ± 21.8 (0.3–152)	<0.01

Data are displayed as mean ± standard deviation and range.

**Table 3:** Anatomical distribution of iatrogenic postoperative nerve lesions in dependence on performed surgery.

	Localization of Iatrogenic Nerve Injury					Total
	Median Nerve	Radial Nerve	Ulnar Nerve	Median + Radial Nerve	Median + Ulnar Nerve	
**PCP (*n*)**	5	0	18	0	1	**24**
**AN (*n*)**	5	4	3	1	1	**14**
***p*-value (Total Lesions PCP vs. AN):**	-	-	-	-	-	**<0.01**
**Total (*n*)**	10	4	21	1	2	**38**

## Data Availability

The datasets used and analyzed during the current study are available from the corresponding author on reasonable request.
